# Endovascular treatment of primary M3 occlusion stroke in clinical practice: analysis of the German Stroke Registry

**DOI:** 10.1186/s42466-024-00330-7

**Published:** 2024-07-18

**Authors:** Niklas M. Beckonert, Johannes M. Weller, Anna C. Alegiani, Tobias Boeckh-Behrens, Milani Deb-Chatterji, Gerhard F. Hamann, Lars U. Krause, Nils C. Lehnen, Louisa Nitsch, Sven Poli, Christian Riedel, Steffen Tiedt, Sarah Zweynert, Gabor C. Petzold, Franziska Dorn, Felix J. Bode, on behalf of the GSR-ET investigators, J Berrouschot, G Bohner, J Borggrefe, A Bormann, M Braun, B Eckert, U Ernemann, MS Ernst, J Fiehler, C Gerloff, K Gröschel, J Hattingen, KH Henn, F Keil, L Kellert, C Kraemer, J Liman, A Ludolph, R Muehl-Benninghaus, O Nikoubashman, C Nolte, M Petersen, A Reich, J Röther, JH Schäfer, M Schell, P Schellinger, E Siebert, F Stögbauer, G Thomalla, C Trumm, T Uphaus, S Wunderlich

**Affiliations:** 1 Department of Vascular Neurology, University Hospital Bonn, Venusberg Campus 1, 53127 Bonn, Germany; 2 Vascular Neurology Research Group, German Center for Neurodegenerative Diseases (DZNE), Bonn, Germany; 3 Department of Neurology, Asklepios Klinik Altona, Hamburg, Germany; 4 Department of Diagnostic and Interventional Neuroradiology, Klinikum Rechts Der Isar, Technical University Munich, Munich, Germany; 5 Department of Neurology, University Hospital Schleswig Holstein Campus Kiel, Kiel, Germany; 6 Department of Neurology and Neurological Rehabilitation, Bezirkskrankenhaus Günzburg, Günzburg, Germany; 7 Department of Neurology, Klinikum Osnabrück, Osnabrück, Germany; 8 Department of Neuroradiology, University Hospital Bonn, Bonn, Germany; 9 Department of Vascular Neurology, Eberhard-Karls University, Tuebingen, Germany; 10 Department of Neuroradiology, University Medical Center Göttingen, Göttingen, Germany; 11 Institute for Stroke and Dementia Research, Klinikum Der Universität München, Ludwig-Maximilians-Universität LMU, Munich, Germany; 12 Department of Neurology, University Hospital Berlin Charité, Berlin, Germany

**Keywords:** Stroke, Endovascular treatment, Mechanical thrombectomy, MeVo, Clinical outcome

## Abstract

**Background:**

Endovascular treatment (ET) options for acute stroke due to distal middle cerebral artery occlusions are rapidly evolving, but data on outcome and safety are sparse. We therefore performed an analysis of patients undergoing ET for primary M3 occlusions in routine clinical practice in a nationwide registry.

**Methods:**

Patients enrolled between 01/20 and 12/21 in the prospective, multicenter German Stroke Registry-Endovascular Treatment (GSR-ET) were screened for mechanical thrombectomy performed for primary M3 occlusion. We analyzed neurological deficit as measured by the National Institute of Health Stroke Scale (NIHSS), symptomatic intracranial hemorrhage (sICH), thrombectomy technique, successful reperfusion (modified Thrombolysis in Cerebral Infarction [mTICI] score of 2b-3) and functional outcome as measured by the modified Rankin Scale (mRS) at discharge and 90 days.

**Results:**

Out of 5574 patients, 11 patients (0.2%, median age 80 years, 54.5% female) underwent ET for primary M3 occlusion. All patients had pre-admission mRS ≤ 1, median NIHSS on admission was 8, and successful reperfusion was achieved in 6/11 patients (54.5%). While no vasospasm, dissection or perforation was reported, symptomatic intracranial hemorrhage occurred in 2 patients (18.2%). Favorable outcome (mRS ≤ 2) was achieved in 6/11 patients (54.5%) at 90-day follow-up.

**Conclusions:**

ET for primary M3 occlusions is rarely performed. While technically feasible, the procedure’s potential benefits must be carefully weighed against its associated risks, including clinically relevant complications. Caution and further research is needed to optimize patient selection for this intervention.

**Trial Registration:**

GSR-ET; ClinicalTrials.gov Identifier: NCT03356392; Trial Registration Date: 11/29/2017.

**Supplementary Information:**

The online version contains supplementary material available at 10.1186/s42466-024-00330-7.

## Introduction

Endovascular treatment (ET) has become the standard of care for acute stroke caused by large vessel occlusion (LVO) [1]. Increasing clinical experience and evidence are emerging for ET as a treatment option for distal occlusions of the middle cerebral artery (MCA) [2–4]. According to current guidelines, there is class IIb evidence that ET "may be reasonable" in selected patients with occlusions of the MCA segment 2 or MCA segment 3 [5]. While ET is generally favored over best medical therapy for primary M2 occlusions [4, 6, 7], ET for primary M3 occlusion stroke (M3OS) is more challenging: First, there is an increased risk of complications, such as perforation, dissection and vasospasm, due to the longer route into small-diameter vessels [8]. Second, the neuronal "tissue at risk" is smaller, resulting in a potentially smaller loss of function when compared to proximal occlusions. Third, it remains unclear whether additional ET is beneficial in patients receiving intravenous thrombolysis (IVT) for primary M3OS. On the other hand, primary M3OS can lead to devastating clinical deficits, especially in eloquent areas.

Accordingly, ET in the M3 segment has been performed only in selected cases to date. Small case series have demonstrated the technical feasibility [9–11], but there are no larger case series of primary M3OS, and results of randomized trials comparing ET with best medical therapy are lacking. Therefore, we here aimed to investigate feasibility, safety parameters, and outcomes of M3OS in a large multicenter German thrombectomy registry.

## Methods

We used data from the German Stroke Registry – Endovascular Treatment (GSR-ET; ClinicalTrials.gov identifier: NCT03356392). The GSR-ET is an ongoing, academic, open-label, multicenter registry of consecutive patients with acute ischemic stroke undergoing ET. The study was conducted in accordance with the Declaration of Helsinki and was approved centrally by the Institutional Review Board of the Ludwig-Maximilians-University Munich (689–15) and by local institutional review boards. Detailed methods of the GSR-ET have been published previously [12, 13].

Primary M3 occlusion was defined as proof of occlusion of the MCA distal to the M2/M3 junction and proximal to the M4 junction within the (opercular) M3 segment [14] on initial imaging or peri-interventional proof of M3 occlusion on angiography. Secondary occlusions such as migrated proximal LVO, peri-interventionally displaced or fragmented thrombus material, and multilocular occlusions on initial imaging were excluded. The item of M3 occlusion was introduced into the registry database in January 2020. For the current analysis, the registry data were used from the introduction of that item until December 31, 2021. For suspected M3OS cases, the treating centers were asked to re-evaluate the initial imaging and angiography for anatomic location of the MCA occlusion and whether the inclusion criterion of primary M3OS was met. In addition, information on thrombectomy technique, employed device and complications was requested.

Stroke severity was assessed using the National Institutes of Health Stroke Scale (NIHSS), and outcome and premorbid disability were rated by the modified Rankin Scale (mRS). Baseline infarct size was assessed using the Alberta Stroke Program Early CT Score (ASPECTS). Reperfusion success was defined as a modified Thrombolysis in Cerebral Infarction (mTICI) score of 2b-3 [15]. Intracranial hemorrhage (ICH) following stroke and reperfusion therapy was classified according to the Heidelberg Bleeding Classification [16]. Symptomatic intracranial hemorrhage (sICH) was defined as intracranial hemorrhage on 24-h follow-up CT and at least a 4-point increase in NIHSS [17]. Stroke etiology was rated according to the TOAST classification [18].

The neurological endpoints were functional outcome measured by mRS at discharge and at 90-day follow-up [1]. Secondary endpoints were the rate of good functional outcome defined as mRS 0–2 at 90-day follow-up, NIHSS at 24 h and at discharge, periprocedural complications (dissection, perforation, vasospasm), sICH, in-hospital-mortality and mortality at 90-day follow-up.

Missing data were not imputed for this analysis. Standard descriptive statistics were provided. Statistical calculations were performed using R (R version 4.2.1, R core team 2022).

## Results

**Fig. 1:**
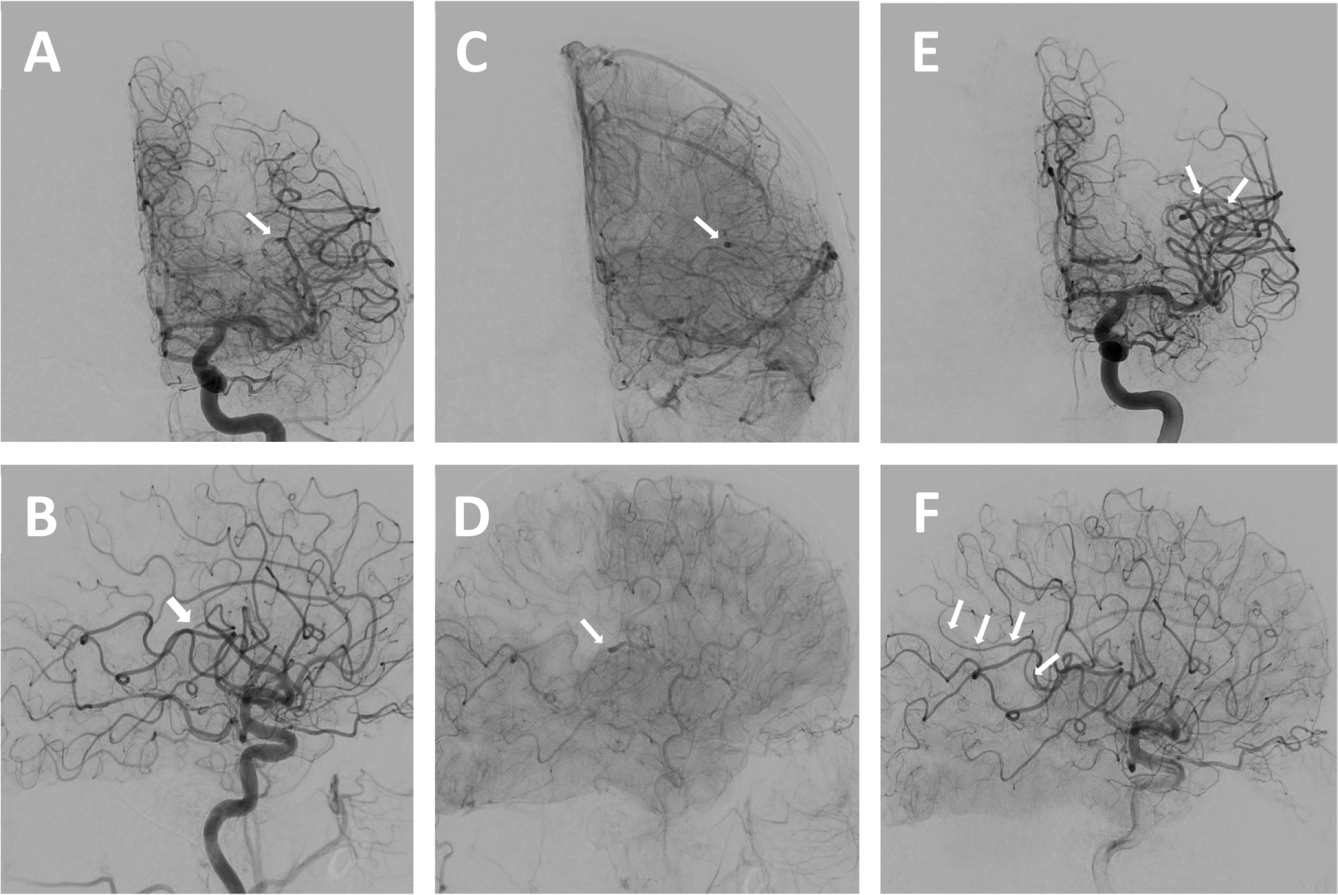
Endovascular treatment for primary M3 occlusion stroke. Initial run (**A** (PA), **B** (lateral): arterial, **C** (PA), **D** (lateral): parenchymal phase) demonstrates M3-occlusion (➔) with parietal perfusion deficit and full reperfusion of the branch ( ➔➔) after stent retriever thrombectomy (**E**, **F**)

A total of 5574 patients treated with ET were included in the registry between January 2020 and December 2021. Of those, M3 occlusion was documented in 57 cases and isolated M3 occlusion was identified in 20 cases from 9 centers (Supp. Figure 1). After re-evaluation of these cases, 11 were confirmed to have primary M3 occlusion (example shown in Fig. 1). Thus, primary M3OS accounted for 0.2% of all ETs performed.

The median age was 80 years (interquartile range (IQR), 65.5–85.5) and 54.5% were female (Table 1). All patients were functionally independent prior to treatment (mRS ≤ 1) and 63.6% were fully independent (mRS 0). The median NIHSS score was 8 (IQR, 6.5–11) and the median ASPECT score was 10 (IQR, 9–10). Interestingly, the occluded vessel was located in the left hemisphere in all cases.

**Table 1:** Main baseline and outcome characteristics of patients undergoing endovascular treatment for primary M3 occlusion stroke

	Age	Sex	Pre-morbid mRS	NIHSS	ASPECTS	Occluded vessel Initial Imaging	Side	IVT	Occluded vessel DSA	M3 branch	Final mTICI	sICH	Etiology	Discharge mRS	d90 mRS
*1*	87	m	1	10	10	M3	left	no	prox. M3	Truncus-superior	0	no	cardioembolism	4	4
*2*	88	f	0	14	10	M3	left	yes	M3 and A. pericallosa	NA	0	no	cardioembolism	5	5
*3*	67	f	1	12	9	distal M2	left	no	M3	Parietal	2b	no	cardioembolism	2	2
*4*	64	f	0	6	9	M2/M3 junction	left	yes	M2/M3 junction	Parietal	0	yes	cardioembolism	6	6
*5*	80	m	1	3	10	none	left	no	M3	Truncus superior	2a	yes	paroxysmal nocturnal hemoglobinuria	4	6
*6*	77	m	0	3	10	M3	left	no	M2/M3 junction	Fronto-parietal	3	no	undetermined	2	3
*7*	55	m	0	7	10	prox. M2	left	yes	prox. M3	Parietal	3	no	undertermined	NA	0
*8*	88	f	0	12	10	prox. M3	left	yes	prox. M3	Parietal	2b	no	cardioembolism	1	1
*9*	80	m	0	4	10	M3	left	yes	M2/M3 junction	Parietal	0	no	embolisation from M1 aneurysm	3	2
*10*	84	f	1	8	10	M3	left	no	M3	Parietal	3	no	cardioembolism	2	1
*11*	32	f	0	7	10	M3	left	yes	M3	Frontal	3	no	undetermined	1	0

*Abbreviations*: *ASPECTS* Alberta Stroke Program Early CT Score, *NA* not available, *NIHSS* National Institutes of Health Stroke Scale, *IVT* intravenous thrombolysis, *mTICI* modified Thrombolysis in Cerebral Infarction score, *mRS* modified Rankin Scale, *sICH* symptomatic intracranial hemorrhage

Eight of 11 patients (72.7%) underwent ET under general anesthesia. The median number of thrombectomy maneuvers was 1 (IQR, 1–2). Successful reperfusion (i.e., mTICI score ≥ 2b) was achieved in 6 out of 11 cases (54.5%). All patients were treated at larger centers, recruiting between 167 and 212 cases at the respective year.

No complications in terms of perforation, vasospasm or device malfunction were reported. In a single case, there was a clot migration into the distal vessels. 3 cases showed class 3c subarachnoid hemorrhage (SAH). sICH occurred in 2 cases, and both patients eventually died. 6 of 11 patients (54.5%) received IVT. One additional case not receiving IVT due to prior anticoagulation was periprocedurally treated with 10 mg of rtPA i.a. due to incomplete recanalization. Complete recanalization was not achieved (mTICI 2a) and follow-up imaging revealed sICH and SAH.

Stent retriever thrombectomy combined with distal aspiration was the preferred technique, performed in 72.7% of cases (8/11), whereas stent retriever thrombectomy was used in 2 cases and direct aspiration in only 1 case. Small lumen microcatheters and dedicated small stent retrievers were used in all stent retriever cases**.** Details of pre- and periprocedural times and clinical characteristics are given in Table 1 and in the Supplemental Material.

The most common stroke etiology according to the TOAST classification was cardioembolism in 54.5% of cases. Rare causes were paroxysmal nocturnal hemoglobinuria and embolization from an M1 aneurysm, each occurring in one case. Etiology was undetermined in 3 cases.

Functional independence at 90-day follow-up was achieved in 54.5% (6/11) of all patients. Excellent functional outcome (mRS 0–1) was achieved in 4 cases (36.4%). In-hospital mortality was 9.1% (1/11) with one additional death during follow-up. Of note, successful reperfusion was not achieved in either of the deceased patients.

## Discussion

Our case series, reporting on patients with primary M3OS, provides insight into the real-world application of ET in a challenging setting. ET for primary M3OS is rarely performed, accounting for only 0.2% in our registry. While the incidence of primary M3OS is unknown, distal vessel occlusions represent 20–40% of strokes [8], suggesting that only highly selected patients underwent ET in our large nation-wide registry representing approximately 16% of all ET in Germany during the study period [19]. Our data show a clear trend in the selection of eligible patients for ET for primary M3OS: These patients were functionally independent prior to stroke, clinically severely affected and had no or only little infarct demarcation on pre-interventional imaging. Exclusively left hemispheric occlusions combined with high NIHSS scores suggested that eloquent branches were affected, leading to the clinical decision that potential benefits outweighed risks in these cases.

The feasibility of ET in primary M3OS is emphasized by the rate of successful recanalization and functional independence at follow-up, which was achieved in both 55%. Final mTICI score ≥ 2b was observed in 5 out of 6 patients who achieved a good clinical outcome (mRS 0–2) at 90 days. This may suggest that prompt recanalization remains the key factor in patients’ functional improvement in distal/medial vessel occlusions, as previously demonstrated in LVO strokes. Notably, recanalization rates were much lower than those observed in large studies of proximal vessel occlusion stroke [1]. Although only experienced high-volume centers were involved in this study, there seems to be a relevant safety risk in performing ET for primary M3OS as subarachnoid hemorrhage was observed in almost 30% of cases. We speculate that the longer and more tortuous route of the catheter into the small and often winding M3 segment may have led to perforation, as perforation is twice as likely in medium and distal occlusions compared to large vessel occlusion thrombectomy [20].

The risk–benefit balance of ET in M3OS also depends on the administration of IVT: When IVT is administered, clinicians tend to omit ET for distal vessel occlusions [21]. This practice is supported by higher recanalization rates of IVT in distal occlusions than in proximal occlusions [22]. However, contrary to previous assumptions, Saver et al. highlighted that IVT in distal vessel occlusions results in only 30–50% resolution of the visualized thrombi [8]. Therefore, medium and distal vessel occlusions are the next frontier of ET and highly clinically relevant, as they account for 20–40% of all strokes [8].

Increasing technical innovations such as microstent retrievers and the use of smaller and potentially more flexible microcatheters constantly expand the indications for and the overall technical success of ET. Accordingly, difficult discussions about performing ET for primary M3 occlusions will increase in the clinical routine.

In addition to the limitations inherent to a retrospective multicentric cohort study, the main limitation is the low number of included M3OS patients despite our registry representing approx. 16% of all ET performed in Germany during the study period [19]. Further, as only 0.2% of patient in the GSR-ET underwent ET for M3OS, it is not possible to make a statement on patient outcomes based on this highly selected population, and there was no control group of patients not undergoing ET. Further research is warranted to fully understand the efficacy and safety of ET for M3OS. The ESCAPE MeVO (NCT05151172), DISCOUNT (NCT05030142), DISTALS (NCT05152524), and DISTAL (NCT05029414) randomized controlled trials comparing ET for medium and distal vessel occlusion stroke with best medical therapy are currently recruiting. These trials will hopefully provide important decision support for ET in distal MCA occlusion stroke. Until then, ET for primary M3 occlusion requires a thoughtful risk–benefit analysis.

## Conclusion

Thrombectomy for isolated M3 occlusion stroke is technically feasible, albeit rarely performed. Its potential benefits should be judiciously balanced against its inherent risks, including the occurrence of clinically relevant complications. Prudent patient selection and refinement of interventional devices are imperative to optimize treatment outcomes. Further collaborative research, encompassing large-scale randomized controlled trials, is warranted to refine the selection criteria for distal middle cerebral artery thrombectomy in clinical practice.

### Supplementary Information

Supplementary Material 1: Supplementary Table 1. Baseline, periprocedural and outcome characteristics of patients undergoing endovascular treatment for primary M3 occlusion stroke.

Supplementary Material 2: Supplementary Table 2. Endovascular treatment characteristics of patients with primary M3 occlusion stroke.

Supplementary Material 3: Supplementary Fig. 1. Flow diagram for patient inclusion.

## Data Availability

The data supporting the findings of this study are available from the corresponding author upon reasonable request.

## Abbreviations

ASPECTS: Alberta Stroke Program Early CT Score
ET: Endovascular treatment
IVT: Intravenous thrombolysis
GSR-ET: German Stroke Registry – Endovascular Treatment
LVO: Large vessel occlusion
M3OS: Primary M3 occlusion stroke
MCA: Middle cerebral artery
mRS: Modified Rankin Scale
mTICI: Modified Thrombolysis in Cerebral Infarction
NIHSS: National Institutes of Health Stroke Scale
sICH: Symptomatic intracranial hemorrhage

## References

[1] Goyal M, Menon BK, Van Zwam WH (2016). Endovascular thrombectomy after large-vessel ischaemic stroke: a meta-analysis of individual patient data from five randomised trials. Lancet.

[2] Ospel JM, Goyal M (2021). A review of endovascular treatment for medium vessel occlusion stroke. Journal of NeuroInterventional Surgery.

[3] Herzberg M, Dorn F, Trumm C (2022). Middle cerebral artery M2 thrombectomy: safety and technical considerations in the German Stroke Registry (GSR). Journal of Clinical Medicine.

[4] Sarraj A, Parsons M, Bivard A (2022). Endovascular thrombectomy versus medical management in isolated M2 occlusions: pooled patient-level analysis from the EXTEND-IA Trials, INSPIRE, and SELECT Studies. Annals of Neurology.

[5] Powers WJ, Rabinstein AA, Ackerson T (2019). Guidelines for the early management of patients with acute ischemic stroke: 2019 update to the 2018 guidelines for the early management of acute ischemic stroke: a guideline for healthcare professionals from the American Heart Association/American Stroke Association. Stroke.

[6] Loh, E.D.W., Toh, K.Z.X., Kwok, G.Y.R., et al. (2022). Endovascular therapy for acute ischemic stroke with distal medium vessel occlusion: a systematic review and meta-analysis. *Journal of NeuroInterventional Surgery*. 0:jnis-2022-019717. 10.1136/JNIS-2022-019717 10.1136/jnis-2022-019717 36539273

[7] Mitchell PJ, Yan B, Churilov L (2022). Endovascular thrombectomy versus standard bridging thrombolytic with endovascular thrombectomy within 4·5 h of stroke onset: an open-label, blinded-endpoint, randomised non-inferiority trial. Lancet.

[8] Saver JL, Chapot R, Agid R (2020). Thrombectomy for distal, medium vessel occlusions. Stroke.

[9] Lemmens R, Hamilton SA, Liebeskind DS (2016). Effect of endovascular reperfusion in relation to site of arterial occlusion. Neurology.

[10] Grossberg JA, Rebello LC, Haussen DC (2018). Beyond large vessel occlusion strokes: distal occlusion thrombectomy. Stroke.

[11] Haussen DC, Al-Bayati AR, Eby B (2020). Blind exchange with mini-pinning technique for distal occlusion thrombectomy. Journal of NeuroInterventional Surgery.

[12] Alegiani AC, Dorn F, Herzberg M (2019). Systematic evaluation of stroke thrombectomy in clinical practice: The German Stroke Registry Endovascular Treatment. International Journal of Stroke.

[13] Wollenweber FA, Tiedt S, Alegiani A (2019). Functional outcome following stroke thrombectomy in clinical practice. Stroke.

[14] Gibo H, Carver CC, Rhoton AL, Lenkey C, Mitchell RJ (1981). Microsurgical anatomy of the middle cerebral artery. Journal of Neurosurgery.

[15] Zaidat OO, Yoo AJ, Khatri P (2013). Recommendations on angiographic revascularization grading standards for acute ischemic stroke. Stroke.

[16] Von Kummer R, Broderick JP, Campbell BCV (2015). The Heidelberg bleeding classification. Stroke.

[17] Larrue V, Von Kummer R, Müller A, Bluhmki E (2001). Risk factors for severe hemorrhagic transformation in ischemic stroke patients treated with recombinant tissue plasminogen activator. Stroke.

[18] Adams HP, Bendixen BH, Kappelle LJ (1993). Classification of subtype of acute ischemic stroke definitions for use in a multicenter clinical trial. Stroke.

[19] Ungerer MN, Bartig D, Richter D, Krogias C, Hacke W, Gumbinger C (2024). The evolution of acute stroke care in Germany from 2019 to 2021: Analysis of nation-wide administrative datasets. Neurological Research and Practice.

[20] Schulze-Zachau, V., Brehm, A., Ntoulias, N., et al. (2023). Incidence and outcome of perforations during medium vessel occlusion compared with large vessel occlusion thrombectomy. *Journal of NeuroInterventional Surgery*. 0:jnis-2023–020531. 10.1136/JNIS-2023-020531 10.1136/jnis-2023-020531 37524518

[21] Goyal M, Ospel JM, Menon BK, Hill MD (2020). The next frontier?. Journal of NeuroInterventional Surgery.

[22] Seners P, Turc G, Maïer B, Mas JL, Oppenheim C, Baron JC (2016). Incidence and predictors of early recanalization after intravenous thrombolysis: a systematic review and meta-analysis. Stroke.

