# Supplementary material for: Endovascular treatment of primary M3 occlusion stroke in clinical practice: analysis of the German Stroke Registry
Source: Neurol Res Pract. 2024 Jul 18;6:36. doi: 10.1186/s42466-024-00330-7 (PMC11256396; doi:10.1186/s42466-024-00330-7)
Supplement: Supplementary file 1 — Supplementary Material 1: Supplementary Table 1. Baseline, periprocedural and outcome characteristics of patients undergoing endovascular treatment for primary M3 occlusion stroke. [file 42466_2024_330_MOESM1_ESM.docx]

**Supplementary Table 1: Baseline, periprocedural and outcome characteristics of patients undergoing endovascular treatment for primary M3 occlusion stroke**

*Abbreviations: ASPECTS, Alberta Stroke Program Early CT Score; IQR, interquartile range; NIHSS, National Institutes of Health Stroke Scale; IVT, intravenous thrombolysis; mTICI, modified Thrombolysis in Cerebral Infarction score; mRS, modified Rankin Scale; SO, symptoms onset; LSW, last seen well; ADM, admission; GRO, groin puncture; FLR, flow restoration; sICH, symptomatic intracranial hemorrhage.*

|  | **Primary M3 occlusion**  **n=11** |
| --- | --- |
| Median age, years (IQR) | 80 (65.5-85) |
| Female, % (n) | 54.5 (6) |
| Median prestroke mRS (IQR) | 0 (0-1) |
| Median NIHSS (IQR) | 8 (6.5-11) |
| Median ASPECTS (IQR)  After matching | 10 (9-10) |
| Cardiovascular risk factors, % (n) |  |
| dyslipidemia | 36.4 (4) |
| diabetes mellitus | 18.2 (2) |
| hypertension | 36.4 (4) |
| atrial fibrillation | 45.5 (5) |
| current smoking | 18.2 (2) |
| Baseline medication, % (n) |  |
| Antiplatelet therapy | 9.1 (1) |
| Oral anticoagulation | 9.1 (1) |
| Periprocedural results |  |
| IVT, % (n) | 54.6 (6) |
| mTICI ≥ 2b, % (n) | 54.6 (6) |
| Passages, n (IQR) | 1 (1-2) |
| Median time SO to ADM (IQR) | 88 (76-94) |
| Witnessed onset, % | 77.8 (7) |
| Median time ADM to GRO (IQR) | 77 (60-94) |
| Median time GRO to FLR (IQR) | 28 (26-42) |
| Hospital stay |  |
| sICH after 24h, % (n) | 18.2 (2) |
| Median NIHSS at 24h (IQR) | 7 (4-10.5) |
| Median discharge NIHSS (IQR) | 4 (1-5) |
| Median discharge mRS (IQR) | 2.5 (2-4) |
| Mortality at discharge, % (n) | 10.0 (1) |
| Median duration of stay, d | 12 (7-14) |
| Etiology, % (n) |  |
| Cardioembolism | 54.5 (6) |
| Dissection | 0 (0) |
| Large artery atherosclerosis | 0 (0) |
| Other determined etiology | 18.2 (2) |
| Undetermined etiology | 27.3 (3) |
| Outcome |  |
| Median mRS at 90-day (IQR) | 2 (1-4.5) |
| mRS 0-2 at 90-day, % (n) | 54.5 (5) |
| mRS 0-1 at 90-day, % (n) | 36.4 (4) |
| Mortality at 90-day, % (n) | 18.2 (2) |
|  |  |
|  |  |
