# Supplementary material for: Endovascular treatment of primary M3 occlusion stroke in clinical practice: analysis of the German Stroke Registry
Source: Neurol Res Pract. 2024 Jul 18;6:36. doi: 10.1186/s42466-024-00330-7 (PMC11256396; doi:10.1186/s42466-024-00330-7)
Supplement: Supplementary file 2 — Supplementary Material 2: Supplementary Table 2. Endovascular treatment characteristics of patients with primary M3 occlusion stroke. [file 42466_2024_330_MOESM2_ESM.docx]

**Supplementary Table 2: Endovascular treatment characteristics of patients with primary M3 occlusion stroke**

*Abbreviations: ICH, intracranial hemorrhage*

|  | anesthesia | technique | microcatheter | passages (n) | ICH (Heidelberg bleeding classification) |
| --- | --- | --- | --- | --- | --- |
| **1** | general | Attempt stent retrieval and aspiration thrombectomy | Multiple frustrated attempts to position the 3 Max microcatheter proximal to the thrombus. Switch to 5F Sofia and Headway Duo. | 2 | no |
| **2** | local | Attempt stent retrieval and aspiration thrombectomy | Headway 21 | 0 | 3c |
| **3** | general | Asp (5F)+Aperio Hybrid 2.5/16mm. | Neuroslider 017 | 1 | no |
| **4** | general | Asp (5F)+Aperio Hybrid 2.5/16mm. | Neuroslider 017 | 5 | 2 (PH2) |
| **5** | local | Tiger-Retriever13-System | Echelon-10 | 2 | 2 (PH2) + 3c |
| **6** | local | 6F Sofia + Aperio Hybrid 3,5/28 Stents | Neuroslider 17 | 1 | no |
| **7** | general | Asp (5F) + Aperio3,5x28 | Neuroslider 17 | 1 | no |
| **8** | general | Asp (5F) + Asp 3Max | 3Max | 1 | no |
| **9** | general | CR: pRESET | Rebar 18 | 2 | NA |
| **10** | general | CR: Catch mini view | Echelon-10 45 Grad tip shape | 1 | 3c |
| **11** | general | CR: Catch view mini | Rebar 18 | 1 | NA |
