# Supplementary material for: Endovascular treatment of primary M3 occlusion stroke in clinical practice: analysis of the German Stroke Registry
Source: Neurol Res Pract. 2024 Jul 18;6:36. doi: 10.1186/s42466-024-00330-7 (PMC11256396; doi:10.1186/s42466-024-00330-7)
Supplement: Supplementary file 3 — Supplementary Material 3: Supplementary Fig. 1. Flow diagram for patient inclusion. [file 42466_2024_330_MOESM3_ESM.docx]

**Supplementary Material**

**Supplementary Figure 1:** Flow diagram for patient inclusion

excluded due to re-evaluation (n=9; 2 cases were re-evaluated as more proximal occlusions, 1 case was concomitant ACI stenting, 1 case was a fragmented thrombus with M3 involvement, 2 cases were more distal occlusions without intervention, 3 cases had incomplete data)

Confirmed primary M3 occlusion (n=11)

Suspected primary M3 occlusion (n=20)

M3 occlusion (n=50)

Reported M3 information 01/20-12/21 (n=5,574)

before systematic evaluation of M3 (n=5442), no information on M3 (n=255)

GSR-ET patients with anterior circulation LVOS (n=11,271)

posterior circulation (n=1429), no data available (n=382)

GSR-ET patients (n=13,082)

not isolated M3 occlusion (n=30)

occlusion other than M3 (n=5524)
